# Correspondence

**Published:** 1874-10

**Authors:** 


### Correspondence.

We have received the following letter to which we would reply that we know of no means by which our correspondent could become a member of the Buffalo Medical Association, or send communications to it, unless he was elected a corresponding member. As to the publication of his cases in the daily papers, we can give him no information, except to say that as a matter of courtesy and by request in some cases, we furnish copies of the JOURNAL to the press of the city. We have never seen a report of a case copied from the JOURNAL, but occasionally have seen notices of the contents of a number. If any cases have been copied into the daily papers they have escaped our notice, and we can give no information as to how they came to be copied whether by solicitation of the persons reporting the case or not.

———

UNIONTOWN, N. Y., Oct. 15, 1874.

*Editor Medical Journal:*

I notice that by reporting cases in the Buffalo Medical Association, and publishing in the MEDICAL JOURNAL, they sometimes reach reprint in the *Courier* and, perhaps, other daily papers. I want to inquire if this Society would receive papers from outside physicians, or can physicians from other towns join? I have some cases not in themselves remarkable, but which if published in a newspaper would make at least a good advertisement, and as I am just begining might help start. These cases are common, such as fevers, bowel complaints, some amputations, exsections of joints, one operation for hernia, &c., &c. Please tell the expense of joining the Society, and also if there is any expense in getting the newspapers to copy. Does the Society pay this expense, or does it come out of the author's of reports?

Yours most sincerely,

NEWMAN W. SMITH, M. D.

